# Experimental Enhancement of Feelings of Transcendence, Tenderness, and Expressiveness by Music in Christian Liturgical Spaces

**DOI:** 10.3389/fpsyg.2022.844029

**Published:** 2022-03-10

**Authors:** Samantha López-Mochales, Raquel Jiménez-Pasalodos, Jose Valenzuela, Carlos Gutiérrez-Cajaraville, Margarita Díaz-Andreu, Carles Escera

**Affiliations:** ^1^Brainlab – Cognitive Neuroscience Research Group, Department of Clinical Psychology and Psychobiology, Faculty of Psychology, University of Barcelona, Barcelona, Spain; ^2^Institute of Neurosciences, University of Barcelona, Barcelona, Spain; ^3^Departament d’Història i Arqueologia, Universitat de Barcelona, Barcelona, Spain; ^4^Sección Departamental de Historia y Ciencias de la Música, Universidad de Valladolid, Valladolid, Spain; ^5^Institució Catalana de Recerca i Estudis Avançats (ICREA), Barcelona, Spain; ^6^Institut d’Arqueologia de la Universitat de Barcelona (IAUB), Barcelona, Spain; ^7^Institut de Recerca Sant Joan de Déu (IRSJD), Esplugues de Llobregat, Spain

**Keywords:** psychoacoustics, archaeoacoustics, emotion, music, auralization

## Abstract

In western cultures, when it comes to places of worship and liturgies, music, acoustics and architecture go hand in hand. In the present study, we aimed to investigate whether the emotions evoked by music are enhanced by the acoustics of the space where the music was composed to be played on. We explored whether the emotional responses of western naïve listeners to two vocal pieces from the Renaissance, one liturgical and one secular, convolved with the impulse responses of four Christian temples from the United Kingdom, were modulated by the appropriate piece/space matching. In an alternative forced choice task where participants had to indicate their preference for the original recording of the piece (not convolved with any temple-like acoustics) vs. the convolved one, no significant differences were found. However, in the tasks where participants rated their emotional in response to each piece and acoustic condition, the factorial ANCOVA analyses performed on the results revealed significant effects. We observed that, across pieces and spaces, participants found the temple-like acoustics as more transcendent, compared to the acoustics of the original version of the pieces. In addition, they rated the secular piece as more tender and the liturgical piece as more expressive in its original versions, compared to the convolved ones. We conclude that the acoustic signature of the four Christian temples causes an exaltation of certain emotions on listeners, although this effect is not associated to one or another musical piece.

## Introduction

A growing body of literature has shown the influence of environmental acoustics, including features such as loudness, reverberation and clarity, over listeners’ preference for certain concert halls rather than others (Barron, 1971; Schroeder et al., 1974; Farina et al., 2007; Lokki et al., 2012; Pätynen and Lokki, 2016). Environmental acoustics also drive listeners’ emotional reactions –such as valence and activation– and influence perceptual attributes –such as roughness and sharpness (Västfjäll, 2012).

Despite sound is an ephemeral event, acoustic environments persevere over time (Rainio et al., 2017), so that the acoustic signature of a particular space can be captured with appropriate methodologies by recording its so-called impulse response (IR) (Mattioli et al., 2017; Álvarez-Morales et al., 2020). The IR is a filter that integrates the metrics of sound propagation between a sound emission point and a receiver device located in the same environment (Farina, 2007). Furthermore, by the use of an auralization approach (Vorländer, 2008), which involves a convolution-based reverberation technique (Farina and Ayalon, 2003), it is possible to apply the acoustic characteristics of the space where the impulse response is recorded to any given sound. Auralization allows to immerse listeners in a particular sonic space, to further evaluate their individual reaction, subjective interpretation and affective responses, to ultimately explore the connection between emotions and sound physical properties (Västfjäll et al., 2002; Pätynen and Lokki, 2016).

The preferred amount of reverberation on stage, in case of performing musicians, strongly depends on the piece of music being interpreted, as shown by Woszczyk and Martens (2008). In case of listeners, as shown in the study by Kuusinen in 2014 (Kuusinen et al., 2014), distinct preferences for reverberation, loudness, and clarity are manifested for different musical pieces. This suggests that the physical properties of sound propagation in different buildings are strongly related to the aural intention of the space, leading to the kind of music to be interpreted there. For example, in Western musical cultures, spaces with short reverberation times and high clarity are more suitable for speech and rapid sounds, while long reverberation times are better for interpreting music characterized by slow sound sequences that require blending, such as Christian liturgical music (Scarre and Lawson, 2006). Christian liturgical music has been, throughout different epochs, particularly bond to its performative space, architectures and symbolic constructions, and there are tight linkages between musical compositions and their places of production (Ferrando, 2020).

The main purpose of the present study was to establish a relationship between the acoustic signature of singular spaces, the compositional styles, and the emotions evoked on the listener, with regards to Christian liturgical pieces and constructions. The experimental phase for this study was undertaken in the summer of 2020. We chose two vocal musical pieces from the English Renaissance, one liturgical and one secular, to be convolved with the acoustic data from several English Christian temples –including chapels, churches, and cathedrals– so that participants heard the pieces like being interpreted in those worship spaces. The emotional enhancement of subjects was then examined in order to check whether it was consistent with the specific compositional styles, cultural functions, and historical performative contexts. Finally, we inquired whether, by means of changing the acoustics alone, listeners showed general preferences and if those could be linked to the specific compositional styles and cultural meaning of each piece.

The study tested the hypothesis that emotional responses evoked on listeners after listening to the original studio recordings of the musical pieces will exhibit differences with those evoked after listening to the piece modified, through convolution with impulse responses, to approach the acoustics of one or another Christian temple. Furthermore, we expected this difference to have an opposite direction while listening to the liturgical piece –originally composed to be performed in medium-sized chapels– versus the secular piece –initially created to be performed in private aristocratic homes. Finally, we expected listeners to display a greater preference, in general terms, for the liturgical piece convolved with temple-like acoustics –rather than in its original studio version–, and the other way around for the secular musical piece.

## Materials and Methods

### Participants

The online study performed used the Psychstudio^1^ platform for behavioral experiments. The study was conducted during the peak of the COVID-19 Spain’s second wave period (July to September, 2020), which strongly hindered the recruitment of participants for face-to-face experiments while ensuring all the health safety measures. This led us to opt for the online format. The study was approved by the Bioethics Committee of the University of Barcelona. All methods were performed in accordance with the relevant guidelines and regulations. All participants gave written informed consent in compliance with the Code of Ethics of the World Medical Association (Declaration of Helsinki).

The study consisted of two separate experiments, Experiment A (mandatory) and Experiment B (optional for those participants who, after Experiment A, were willing to continue). The objective of Experiment A was to assess participants’ emotional reactions to musical pieces as if listened in different sonic spaces. The objective of Experiment B was to assess participants’ general preference for the musical pieces used in Experiment A as heard in original versus convolved with temple-like acoustics. Forty-nine individuals –24 women, 24 men, and one person who preferred not to report their gender, of ages between 20 and 33– participated in Experiment A. Of these, 26 decided to participate also in Experiment B. Each individual confirmed, via a questionnaire, being older than 18 and younger than 35 years, having no auditory impairment, not suffering from any neurological or psychiatric diseases, and taking no medication that may affect the central nervous system. Those who did not fulfill these conditions were excluded as participants. Although we cannot ensure the fulfillment of the requirements, participants were asked to use headphones –not speakers– for the experiments, to carry them out in an environment as quiet as possible, and to adjust the volume of their devices to a comfortable level. In addition, participants were presented with a test signal that consisted in one of the excerpts that they would listen to later during the study, randomly chosen, and asked to adjust the volume of their devices to a comfortable level, and to keep it unmodified during the study.

Before starting the experiment, participants were asked to fill a questionnaire about their religiosity and spirituality [the Centrality of Religiosity Scale (Huber and Huber, 2012)], and a questionnaire about their musical studies and experience (labeled as Musical background, details available in Table 1). On the basis of the individual, we calculated an index of *religiosity* (ranging 0–28) and an index of *musical background* (ranging 0–15). These indexes were included in the analysis to consider the possible bias introduced by participants’ relation with music and religion or spirituality. This was because we were using, for stimuli, music not necessarily relevant for all listeners across different cultures, to which certain religious character can be attributed. Furthermore, the impulse responses selected were from Christian temples, and individuals used to assist to these environments could attribute them spiritual content as well. Participants were also asked to report if they professed a religion in particular; all participants reported to be Christian or of any religion, so this item was not considered a possible bias introducer. Therefore, it was not taken into account for the analysis.

**TABLE 1:** Musical background questionnaire.

Question	Possible responses	Punctuation
For how many years have you received musical education (official or not official)?	More than 15 years 10–15 years 5–10 years Less than 5 years None	4 3 2 1 0
Do you dance?	Yes No	3 0
Do you sing or play an instrument?	Yes No	3 0
Since when haven’t you danced/sung/played your instrument?	Today This week This month 1–11 months 1–5 years More than 5 years	5 4 3 2 1 0

*The index of musical background was calculated adding the values corresponding to the answer to each one of the questions.*

### Musical Compositions

The two musical compositions selected purposely fitted geographically and chronologically with the chosen acoustic environments, while still being musically accessible to modern audiences. In this sense, we discarded earlier relevant composers such as Robert Fayrfax, William Mundy and John Browne due to their structural complexity and counterpoint style, which could potentially interfere with contemporary esthetics perception. Consequently, we picked two relatively later pieces, one secular and one liturgical, who displayed a good balance between historical and transhistorical features. Since we aimed to compare between two versions of the same song, and not to perform comparisons of the songs with one another, we did not take into account weather listeners made a conscious discrimination between the two musical genres or not.

English composer William Byrd’s *Ave Verum Corpus* (first published in his book *Gradualia*, 1605) was the liturgical piece used in the present experiment. Originally composed to fit a medium reverberant space, *Ave Verum Corpus* is a hymn that refers to the Catholic belief in transubstantiation. The book is dedicated to both Henry Howard and Byrd’s patron, Sir John Petre, a wealthy Catholic nobleman who, due to the difficult situation faced by practicing Catholics in the late 16th and early 17th centuries, performed Catholic rites clandestinely. Therefore, *Ave verum corpus* was originally composed to be performed in a medium-sized chapel. However, the piece has become a classic of choral music and, from the 19th century onward, has been performed in large temples due to, above all, the revival of Renaissance music. Today, *Ave verum corpus* is a fundamental musical piece not only for Catholic worship, but also in Anglican liturgy. It is one of the most important pieces of European choral repertoire, and thus, has also been commonly played, throughout the centuries, in medium and large reverberant spaces (Kerman, 1981). Musically, Byrd creates a very expressive homophonic declamation, which unfolds great sonic richness through short phrases and clear-cut cadences, in order to favoring the clarity and expression of the text. The second composition, *Weep, oh mine eyes*, by English composer John Bennet (printed in a book entitled *Madrigals to Four Voices*, 1599), is a well-known madrigal, a secular music composition of the Renaissance, which was not conceived to be performed in a very reverberant space. Usually, this type of refined secular compositions was sung in private chambers of noble houses. In principle, a large reverberant space would affect the intelligibility and independence of the voices. Unlike Byrd’s work, in which the relationship between the four voices is conceived vertically, in Bennet’s piece the vocal lines are intertwined in an imitative counterpoint of great beauty and expressiveness. The motivic work made by Bennet in each of the four voices is precise and clearly fits the emotional intention of the composition, which quickly envelops the listener in a sad and melancholic atmosphere.

### Stimuli

Two stimulus sets were assembled, set A –employed in Experiment A– and set B –employed in Experiment B. To assemble set A, 10 excerpts of 30 s of duration were extracted from the two musical pieces (five excerpts from each piece): the liturgical piece *Ave Verum Corpus* by William Byrd (1543–1623) interpreted by the vocal sextet The King’s Singers (1995, 2012), available in the album *English Renaissance* (1995), and the secular piece *Weep, oh mine eyes* by John Bennet (1575–1614), interpreted by the same vocal sextet and available in the album *Royal Rhymes and Rounds* (2012).

From the liturgical piece *Ave Verum Corpus*, we selected five excerpts that represented all the parts of the piece, discarding the repetition (*O dulcis pie*) and including the coda. In the case of the secular piece *Weep oh mine eyes* the five excerpts of 30 s covered almost the whole duration of the recording.

The set A of stimuli consisted of these 10 excerpts presented in two acoustic conditions: in its original studio recording version (which will be referred to, from now on, as raw) and convolved with an impulse response from a Christian temple (which will be referred to, from now on, as convolved). Therefore, 20 excerpts in total conformed set A. To assemble set B, 10 excerpts of 10 s of duration were extracted from the same two musical pieces (five excerpts from each piece). The set B of stimuli consisted of 20 pairs of excerpts: each one of the mentioned 10 excerpts presented first in raw condition, and then in convolved condition, and the other way around (first in convolved condition and then in raw condition).

**FIGURE 1:**
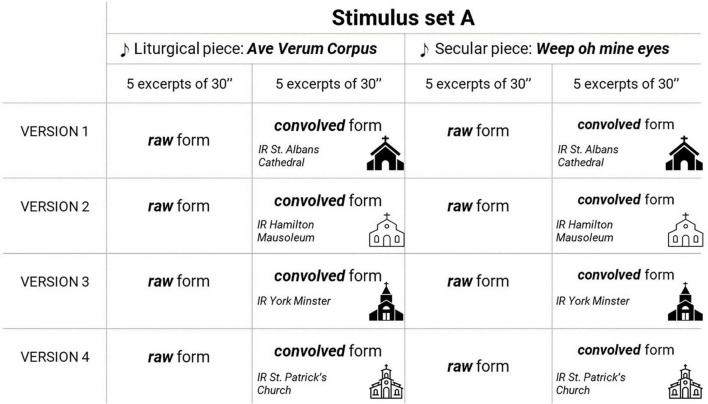
Schema of stimulus set A and its versions.

**FIGURE 2:**
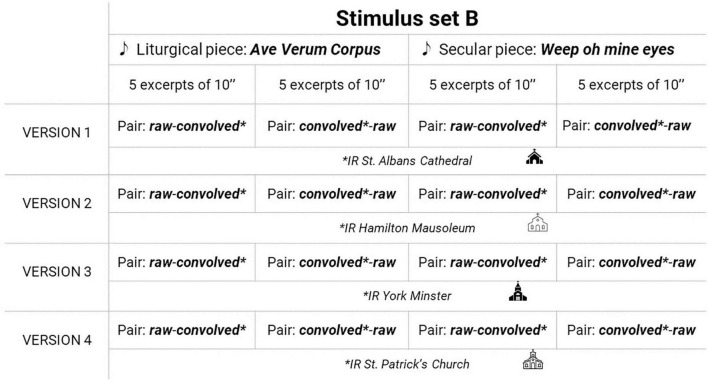
Schema of stimulus set B and its versions.

Four impulse responses were used to assemble the convolved excerpts of the set A and set B of stimuli, generating four versions of each set. The impulse responses were recorded in the Lady Chapel of St Albans Cathedral (St Albans, United Kingdom), the Hamilton Mausoleum (Hamilton, United Kingdom), the York Minster (York, United Kingdom), and the St Patrick’s Church (Patrington, United Kingdom). All were publicly available in the Open Acoustic Impulse Response (Open AIR) Library by University of York^2^. A schema of stimulus sets assembling is presented in Figures 1, 2. According to the data provided by the OpenAIR (2020) Open AIR library, the reverberation times (T30) for the four spaces mentioned were 2.3, 11.9, 7.8, and 1.9 s respectively, retrieved by averaging the octave bands from 31.25Hz to 16kHz.

The convolutions of the musical excerpts with the four impulse responses were performed using the software Bidule (2020), by Plogue Art et Technologie, and the X-MCFX plugin, version 0.5.11 (X-MCFX Vst plugin, version 0.5.11, 2014). The format of the original audio files was stereo WAV, and the format of the impulse responses was binaural. After the convolution, the resulting audio files were stored in stereo WAV format as well.

Each participant was randomly assigned to listen to one of the four versions of the set A and set B of stimuli. Eight participants listened to the first version of set A (corresponding to the impulse response from the Lady Chapel of St Albans Cathedral), and of these, seven took part also in Experiment B –and, therefore, listened to the first version of set B. Twelve participants listened to the second version of set A (with the impulse response from the Hamilton Mausoleum), and of these, six took part also in Experiment B. Fifteen listened to the third version of set A (with the impulse response from the York Minster), and of these, nine took part also in Experiment B. Finally, fourteen listened to the fourth version of set A (with the impulse response from the St Patrick’s Church), and of these, five took part also in Experiment B.

All stimuli were presented binaurally in stereo format, known to elicit stronger emotions than monophonic sounds (Västfjäll, 2003).

### Experiment A

In Experiment A, participants listened to the whole set A of 20 musical excerpts of 30 s of duration, presented in random order. To assess the emotions evoked by these stimuli, participants were instructed to rate four visual analog scales (on visual-analog sliding bars, subsequently quantified from 0 - leftmost, to 1000 - rightmost). The first two adjectives were expressiveness (leftmost = what I heard was not expressive at all, rightmost = what I heard was very expressive), and beauty (left = the music was not beautiful at all, to right = the music was very beautiful). Expressiveness, and how it is achieved in performance, has been a dominant topic in music esthetics (Davies, 2001; Juslin and Timmers, 2010). Västfjäll et al., used the rating scale of expressiveness in their study of 2002 where, as in the present work, the effect of non-content features of sound (in particular, reverberation time) on emotional reactions was studied. While the term expression focuses on the reaction of the audience rather than the interpreter (Robinson, 2007), expressiveness is a –not accidental but deliberately created-property of the piece’s sound (Davies, 2001). The next two rating scales were the components of the *circumplex model of affect* (Russell, 1980): arousal (from left = *I felt very calmed, not excited/aroused at all*; to right = *I felt very excited/aroused*) and valence (from left = *I felt no pleasure at all*; to right = *I felt great pleasure*). These items were also used in the study performed by Västfjäll et al. (2002), and are considered pan-cultural (Barrett, 1998), an aspect deem relevant for an online study that was disseminated worldwide.

After rating the four visual analog scales, participants also had to complete the shortest version of the Geneva Emotional Music Scale or GEMS-9, which is specifically designed to evaluate emotions evoked by music. This scale is composed by the following items: Joy, Sadness, Tension, Wonder, Peacefulness, Power, Tenderness, Nostalgia, and Transcendence they felt during the listening, from 1 to 5, being 1 = *Not at all*, and 5 = *Very much* (Zentner et al., 2008).

#### Statistical Analysis

For each of the studied variables (four visual analog scales and nine items conforming the GEMS-9 scale), and for each of the two musical pieces, we performed a factorial ANCOVA analysis. Although the scorings of the GEMS-9 scale are ordinal, they were treated as interval, since rating scales with five or more steps have been confirmed as psychometrically valid (Carifio and Perla, 2007). Parametric methods have been considered robust for the analysis of rating scales results (Norman, 2010) and used in previous studies for the analysis of the GEMS scale scorings (Miu and Rodica-Baltes, 2012; Pearce and Halpern, 2015; Hanser et al., 2016).

The objective of the analysis was to test the effects of the factor *acoustic condition* (with two levels, raw, and convolved), the factor *excerpt* (with five levels, first, second, third, fourth, and fifth), and the factor *version* (with four levels, corresponding to the four impulse responses selected StAlbans, Hamilton, YorkMinster, and StPatricks). The covariates in our model were the indexes of *religiosity* (with values from 0 to 28) and *musical background* (with values from 0 to 15) obtained from the Centrality of Religiosity Scale and the Musical background questionnaire, respectively. The interest was focused on observing the influence of the factor *acoustic condition* over each one of the dependent variables. In all cases, the significance level considered was α = 0.05. All the analyses were carried out using the software R (version 3.6.2; RRID:SCR_001905) and the ez package (Lawrence, 2016; RRID:SCR_020990).

### Experiment B

For experiment B, a different approach was taken in which the general preference of the participants for the raw or convolved condition of the excerpts was interrogated. Participants were presented with each pair of 10-s excerpts, first in raw condition and then convolved with church-like acoustics, and then in the other way around too. Participants had to indicate which excerpt of the pair they preferred, the first or the second.

#### Statistical Analysis

We applied binomial tests to these results, to see if the proportion of responses indicating preference for one or another acoustic condition (raw/convolved) significantly deviated from the expected proportion if participants responded randomly. Significance level considered was α = 0.05. The analysis was carried out using the software R (version 3.6.2; RRID:SCR_001905).

## Results

After analyzing the data obtained from Experiment A, using factorial ANCOVA analyses with the factors *acoustic condition*, *excerpt* and *version*, and the covariates *religiosity* (*M* = 5.90, *SD* = 5.13) and *musical background* (*M* = 8.43, *SD* = 4.01), an influence of the factor of interest (*acoustic condition*) over several variables was found in each one of the two musical pieces. However, no significant results were found after the analysis of the data from Experiment B.

### Experiment A

Regarding the liturgical song, *Ave Verum Corpus*, we spotted a significant effect of the factor *acoustic condition* over the variables Expressiveness and Transcendence. Regarding the secular song, *Weep oh mine eyes*, we observed a significant effect of the factor *acoustic condition* over the variables Tenderness and Expressiveness. The summary of these factorial ANCOVA analyses is presented in Table 2.

**TABLE 2:** Summary of the factorial ANCOVA analyses performed on the variables.

Piece: *Ave Verum Corpus*								
**ANCOVA – DV: Expressiveness**								
**Factors**	**DFn**	**DFd**	**SSn**	**SSd**	**F**	***p*-Value**	***R* ^2^**
*Intercept*	1	45	2.111*10^8^	7.478*10^6^	1270	1.262*10^–34^	0.966
*Version*	3	45	2.60*10^5^	7.478*10^6^	0.521	0.6976	0.034
*Acoustic condition*	1	45	1.064*10^5^	8.436*10^5^	5.678	**0.0215**	0.112
*Excerpt*	4	180	3.146*10^5^	4.241*10^6^	3.338	**0.0115**	0.070
*Version:Ac.Cond*	3	45	9.028*10^3^	8.436*10^5^	0.161	0.9223	0.011
*Version:Excerpt*	12	180	2.035*10^5^	4.241*10^6^	0.720	0.7311	0.046
*Ac.Cond:Excerpt*	4	180	7.196*10^4^	2.542*10^6^	1.274	0.2819	0.028
*Vers.:Ac.Cond:Excerpt*	12	180	1.487*10^5^	2.542*10^6^	0.877	0.5711	0.055
**Piece: *Ave Verum Corpus***							
**ANCOVA – DV: Transcendence**							
*Intercept*	1	45	2.900*10^4^	3.258*10^3^	400.5	4.945*10^–24^	0.899
*Version*	3	45	1.571*10^1^	3.258*10^2^	0.723	0.5434	0.046
*Acoustic Condition*	1	45	4.702	3.626*10^1^	5.836	**0.0198**	0.115
*Excerpt*	4	180	1.150*10^1^	1.427*10^2^	3.627	**0.0072**	0.075
*Version:Ac.Cond.*	3	45	1.642	3.626*10^1^	0.679	0.5694	0.043
*Version:Excerpt*	12	180	1.560*10^1^	1.427*10^2^	1.640	0.0840	0.099
*Ac.Cond:Excerpt*	4	180	6.449*10^–1^	8.431*10^1^	0.344	0.8478	0.008
*Vers.:Ac.Cond:Excerpt*	12	180	6.442	8.431*10^1^	1.146	0.3258	0.071
**Piece: Weep oh mine eyes**							
**ANCOVA – DV: Tenderness**							
*Intercept*	1	45	2.624*10^3^	2.642*10^2^	447.0	5.248*10^–25^	0.909
*Version*	3	45	6.604	2.642*10^2^	0.375	0.7714	0.024
*Acoustic Condition*	1	45	3.265	3.386*10^1^	4.340	**0.0429**	0.088
*Excerpt*	4	180	7.294	1.333*10^2^	2.461	**0.0470**	0.052
*Version:Ac.Cond.*	3	45	6.781*10^–1^	3.386*10^1^	0.300	0.8249	0.020
*Version:Excerpt*	12	180	5.162	1.333*10^2^	0.581	0.8560	0.037
*Ac.Cond:Excerpt*	4	180	2.224	9.379*10^1^	1.067	0.3742	0.023
*Vers.:Ac.Cond:Excerpt*	12	180	1.019*10^1^	9.379*10^1^	1.629	0.0869	0.098
**Piece: Weep oh mine eyes**							
**ANCOVA – DV: Transcendence**							
*Intercept*	1	45	2.939*10^3^	3.529*10^2^	374.7	1.891*10^–23^	0.893
*Version*	3	45	4.020*10^1^	3.529*10^2^	1.709	0.1787	0.102
*Acoustic Condition*	1	45	1.000*10^1^	4.956*10^1^	9.079	**0.0042**	0.168
*Excerpt*	4	180	3.796	1.043*10^2^	1.638	0.1665	0.035
*Version:Ac.Cond.*	3	45	1.838	4.956*10^1^	0.556	0.6467	0.036
*Version:Excerpt*	12	180	7.146	1.043*10^2^	1.028	0.4250	0.064
*Ac.Cond:Excerpt*	4	180	2.041*10^–1^	8.855*10^1^	0.104	0.9811	0.002
*Vers.:Ac.Cond:Excerpt*	12	180	6.848	8.855*10^1^	1.160	0.3153	0.072

*Expressiveness and Transcendence for the liturgical song Ave Verum Corpus, and on the variables Tenderness and Transcendence for the secular song Weep oh mine eyes (results of Part A of the study). DV, dependent variable; DFn, degrees of freedom of the numerator; DFd, degrees of freedom of the denominator; SSn, sum of squares of the numerator; SSd, sum of squares of the denominator.*

**FIGURE 3:**
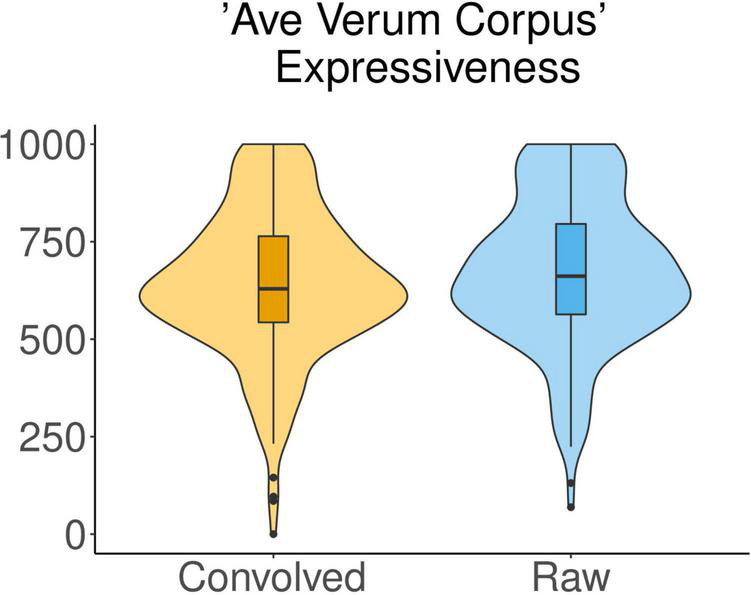
Ratings of Expressiveness for the liturgical piece *Ave Verum Corpus* in raw vs. convolved conditions.

In the liturgical piece *Ave Verum Corpus*, the Expressiveness attributed by participants to the piece was significantly influenced by the *acoustic condition* [*F*(1,45) = 5.678, *p* = 0.0215]. The mean of the values of Expressiveness with which the raw excerpts of this musical piece were qualified (671.8) was higher than the mean of the values for the convolved ones (641.6) (Figure 3). We observed that the factor *excerpt* had some influence over Expressiveness as well [*F*(4,180) = 3.338, *p* = 0.0115] but no significant differences between excerpts were found in *post hoc* pairwise comparisons using *t*-tests.

**FIGURE 4:**
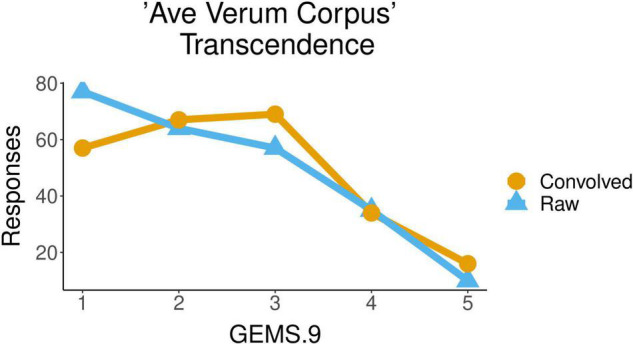
Ratings of transcendence for the liturgical piece *Ave Verum Corpus* in raw vs. convolved condition.

In the same piece, *Ave Verum Corpus*, the Transcendence attributed to the piece by participants was also significantly influenced by the factor *acoustic condition* [*F*(1,45) = 5.836, *p* = 0.0198]. The mean of the values of Transcendence with which the raw excerpts of this musical piece were qualified (2.3) was smaller than the mean of the values for the convolved ones (2.5). Figure 4 shows the distribution of participants’ responses about the transcendence evoked by the piece, with values from 1 to 5, for the two conditions, raw and convolved. We observed that the factor *excerpt* had some influence over Transcendence as well [*F*(4,180) = 3.627, *p* = 0.0072] but no significant differences between excerpts were found in *post hoc* pairwise comparisons using *t*-tests.

**FIGURE 5:**
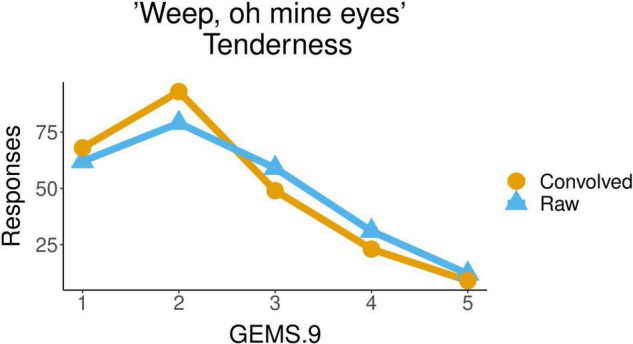
Ratings of tenderness for the secular song *Weep oh mine eyes* in raw vs. convolved condition.

Regarding the secular piece, *Weep oh mine eyes*, the Tenderness attributed by participants to the piece was also significantly influenced by the factor *acoustic condition* [*F*(1,45) = 4.340, *p* = 0.0429]. The mean of the values of Tenderness with which the raw excerpts of this musical piece were qualified (2.4) was higher than the mean of the values for the convolved ones (2.2). Figure 5 shows the distribution of participants’ responses about the tenderness evoked by the piece, with values from 1 to 5, for the two acoustic conditions, raw and convolved. We observed a significant influence of the factor *excerpt* [F(4,180) = 2.461, *p* = 0.0470] but no significant differences were found between excerpts in any *post hoc* pairwise comparisons using *t*-tests.

**FIGURE 6:**
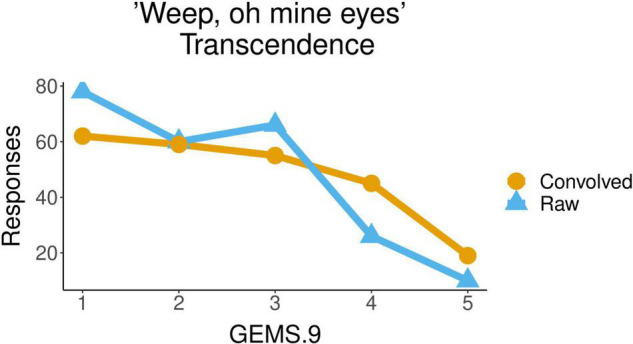
Ratings of transcendence for the secular piece *Weep oh mine eyes* in raw vs. convolved conditions.

Finally, in the case of the secular song *Weep oh mine* eyes also, the Transcendence attributed by participants to the piece was significantly influenced by the *acoustic condition* (F(1, 45) = 9.079, p = 0.0042). The mean of the values of Transcendence with which the raw excerpts of this musical piece were qualified (2.3) was lower than the mean of the values for the convolved ones (2.6). Figure 6 shows the distribution of participants’ responses about the transcendence evoked by the piece, with values from 1 to 5, for the two acoustic conditions, raw and convolved.

### Experiment B

Regarding experiment B, in which participants indicated their overall preference for the raw or convolved form of the musical pieces, no significant differences were found.

## Discussion

The results obtained in our experiment about the emotions elicited by music convolved with the acoustics from Christian temples reveal a significant effect of the acoustic condition over how the Expressiveness in the liturgical piece *Ave Verum Corpus* was scored. Participants qualified the original version of the piece as transmitting higher Expressiveness compared to the same musical piece modified approaching temple-like acoustics. This result was opposite to what we expected, given the musical characteristics of the hymn selected –which benefit from a clear sonority in a sense that homophony prevails over a dense counterpoint in an almost antiphonal texture. Nevertheless, the outcome partially confirms our hypothesis that some dimensions of the music listened are perceived differently depending on the space acoustics.

It is important to indicate that after implementing the experiment we realized that the term Expressiveness caused some confusion among participants. Our intention using this term was to focus on the musical expressiveness emerging from the compositional structure (Schubert and Fabian, 2014) as a measure of how powerful was the piece in terms of transmitting emotions, regardless of the particular emotions perceived (Robinson, 2007). Nevertheless, we later saw that some of our participants may have understood it as referring to the performers’ interpretation of the piece, which is referred, in Schubert and Fabian (2014) as the “performance layer” of musical expressiveness. Toward future experiments, a detailed definition of the terms to rate should be provided to participants, with especial focus on Expressiveness for its difficulty, as it is a “multifaceted construct” (Schubert and Fabian, 2014). It must also be mentioned that music interpreted centuries after it was composed is inevitably modified, intentionally and unintentionally, by the interpreter, and cannot be considered a complete representative copy of the music originally thought by the composer (Woszczyk and Martens, 2008), as the interpreter, even soon after its composition, becomes a participant in the development of the piece (Barrett, 1998).

Looking at the responses for Transcendence, participants considered both musical pieces more transcendent when the temples’ acoustics were virtually applied. We expected a greater exaltation of the feeling of transcendence in the liturgical piece, *Ave Verum Corpus*, convolved with church-like acoustics, considering the spiritual compound of the label, the compositional style and the intentionality of hymns as a way to get closer to God (Ferrando, 2014). However, the results make us notice the relevance of the acoustic envelopment of the music as responsible for the elicitation of transcendence in Western individuals. We must highlight that, although some researchers’ point that the content of auditory stimulation has the biggest impact on emotional reactions rather than non-content features (Genell, 2008), others demonstrate the effect of acoustic characteristics –such as reverberation– on some emotions evoked by music, such as arousal or pleasantness (Västfjäll et al., 2002). It must be highlighted again the possible lack of esthetical relevance of the musical pieces employed for many of the modern-day participants, which probably caused the acoustics to be a more relevant component in emotion exaltation than the content of the musical pieces itself. It is also important to mention that in Western culture, at least today, individuals are used to connect long reverberation time with religious music and sacred spaces (Ghaffari and Mofidi, 2014; Baumann and Niederstätter, 2016).

The influence of the acoustic condition in participants’ feeling of Tenderness has also been observed, but only for the secular piece, *Weep oh mine eyes*. Participants reported higher tenderness when listening to the original recording of the piece, rather than the one convolved with temples’ acoustics. Tenderness may be related to the perceived dimension of the space, and thus, a less reverberant site is assessed as a smaller and more intimate place. However, this was not the case for the secular piece, and consequently, we cannot draw further conclusions.

Despite the selected musical compositions were created to suit, as we have seen, different reverberation times, surprisingly participants did not show, in Experiment B, a particular preference for one or other acoustic conditions presented to them, in any of the two studied musical pieces. Again, we could attribute this to the lack of esthetical relevance of the musical pieces and, therefore, for the match between piece and acoustic space considered correct, for modern-day participants, which could have led to a lack of general preference for one or another version of the pieces.

It should be noted that the impulse responses employed in this study were recorded in the selected spaces years after their construction and well after the time in which the chosen musical pieces were composed to, hypothetically, be interpreted in there. During this long period of time, some elements affecting the acoustics, including ornaments and furniture, may have been removed or replaced (Ferrando, 2020; Álvarez-Morales et al., 2020). More recently, sophisticated solutions to overcome this problem have been put forward, such as those of archaeoacoustics relying on the 3D modeled representations of the sites of interest to simulate the acoustic reflections and virtually obtaining a more accurate impulse response, one that takes into consideration the original structure and materials of both the building and the decoration. An example of this approach was the reconstruction by Manzetti (2016), of the Roman theater of Gortyn, that could not have been acoustically analyzed *in situ* with precision due to its poor preservation.

It must also be mentioned that, to ensure an accurate virtual representation of how would a musical piece sound in certain space by using impulse response reverberation technique, the recording of the piece should be anechoic –containing no sound reflections from the space- or quasi-anechoic. Quasi-anechoic recordings can be obtained with the deconvolution of a recorded signal with the impulse response from the place where it was recorded (Gemba, 2014). These could be especially useful when working with vocal music, as the difficulty of singing in an anechoic environment could worsen the quality of the music. The recordings employed in the present study were not recorded in anechoic conditions, but in a conventional recording studio. Therefore, these recordings may contain some acoustical information from the places where they were recorded, prior to the convolution with the temple-like acoustics. This implies that the convolved version of the pieces may have not provided a completely accurate representation of how would the piece have sounded in the selected spaces. In future experiments, anechoic or quasi-anechoic recordings may be used for the auralization to overcome such inaccuracies.

For the purposes of the present study, we required two musical pieces with very specific features: liturgical and secular vocal music from the English Renaissance to match, chronologically and geographically, with the selected acoustic spaces. Due to the impossibility of performing custom dry recordings of the pieces in anechoic conditions because of the COVID-19 pandemic situation, as we mentioned previously, we could only employ tracks recorded in a regular studio. In order to guarantee a more accurate measurement of the relation between music, acoustics and emotions elicited by their combination, we suggest that greater attention should be paid in future experiments to the preservation of the elements across this workflow, starting with a creation of a proper stimuli set recorded in dry conditions to be sure that the only acoustic modifications introduced are the ones contained in the impulse responses from the desired spaces. Also, a qualitative component introduced in the analysis of the musical pieces could be helpful to better understand the participants’ esthetic journey while listening.

Finally, to separate the cultural component of western music and liturgical spaces from the unbiased emotional reaction that the proper combination of sound and acoustics could elicit, the experiment could be extended to other-than-western listeners. Similar results obtained from participants who, ideally, had never had any contacts with western music and the acoustics from Christian temples, would reinforce the idea that, regardless of the musical composition and what emotions it elicits, the proper match between musical piece and acoustic space maximizes the affective responses. This type of experiment is still to be undertaken.

In this article we have described two experiments designed to investigate the emotional reactions and listeners’ preference for the appropriate match between a musical composition and the acoustic space where the composition was meant to be played on. We employed two musical pieces from the Renaissance, one liturgical and one secular, virtually convolved with the acoustic prints of four Christian temples of different sizes in the United Kingdom. We expected listeners to report stronger feelings and higher preference for the versions of the songs matching with their composition space: the liturgical piece convolved with medium or large-sized temple-like acoustics and the secular piece heard as “raw,” that is, without the convolution with temple-like acoustics. Some significant differences have emerged in the emotions elicited by listening to the raw version of the pieces compared to the convolved version.

Despite the methodological limitations discussed above, some differences were identified in listeners’ emotions elicited during the listening of vocal music from the English Renaissance, with and without the virtual application of the acoustic signatures from Christian liturgical spaces from the United Kingdom. Particularly, Expressiveness, Tenderness, and Transcendence reported by listeners were significantly different while listening to one or another version of the selected musical pieces. However, we could not confirm our initial hypothesis that predicted a stronger emotional reaction for the proper match, in cultural terms, of the musical pieces with its acoustics, probably due to the unfamiliarity of modern-day listeners with the musical esthetics of the time.

Regarding the future prospects for the continuation in this line, employing anechoic or quasi-anechoic recordings for the convolutions would improve the accuracy of the virtual representation of the pieces being interpreted in the spaces of interest. Recruiting other-than-western listeners could reinforce the idea that the proper piece-space match evokes certain emotions in a stronger way regardless of the cultural background. Finally, the use of musical pieces or genres that are relevant for the participants of the experiment, considering their age-gap, could help to overcome the problem of lack of esthetic relevance of the stimuli for the participants.

## Data Availability Statement

The raw data supporting the conclusions of this article will be made available by the authors, without undue reservation.

## Ethics Statement

The studies involving human participants were reviewed and approved by Bioethics Committee of the University of Barcelona. The patients/participants provided their written informed consent to participate in this study.

## Author Contributions

SL-M and CE conceived the study. SL-M, RJ-P, JV, and CG-C designed the experimental approach. SL-M acquired, analyzed, and interpreted the data. All authors contributed to writing and finalized the manuscript.

## Conflict of Interest

The authors declare that the research was conducted in the absence of any commercial or financial relationships that could be construed as a potential conflict of interest.

## Publisher’s Note

All claims expressed in this article are solely those of the authors and do not necessarily represent those of their affiliated organizations, or those of the publisher, the editors and the reviewers. Any product that may be evaluated in this article, or claim that may be made by its manufacturer, is not guaranteed or endorsed by the publisher.

## Footnotes

1 Psychstudio (2019). Psychstudio (Version 2019) [Computer software]. Australia. Retrieved from https://www.psychstudio.com.

2 OpenAIR – Open Air Library. Retrieved 01 July 2020, from https://www.openair.hosted.york.ac.uk/.
